# 髓系肿瘤伴大颗粒淋巴细胞增殖的临床特征研究

**DOI:** 10.3760/cma.j.cn121090-20231219-00327

**Published:** 2024-04

**Authors:** 晨霄 杜, 光朋 向, 岚 彭, 湘遥 肖, 广帅 滕, 宇卉 张, 艳 王, 依璠 段, 宗鸿 邵, 洁 白

**Affiliations:** 天津医科大学第二医院血液科，天津 300211 Department of Hematology, the Second Hospital of Tianjin Medical University, Tianjin 300211, China

## Abstract

髓系肿瘤（MN）为一组造血干祖细胞生物学功能异常的血液系统恶性肿瘤。越来越多的证据表明，MN患者异常的免疫、造血微环境与恶性克隆性造血干细胞相互作用，促进其疾病的发生、发展。髓系肿瘤伴大颗粒淋巴细胞增殖（Myeloid neoplasms-large granular lymphocyte proliferation, MN-LGLP）为此类疾病中的一种特殊罕见的临床现象，目前国内外对本病的队列研究较少，本研究分析该类患者的临床和实验室特征，探讨影响LGLP对MN患者临床特征和生存的影响。提示MN-LGLP患者更易出现中性粒细胞减少及脾大。LGLP的存在不是影响MN-LGLP患者生存的危险因素。STAG、ASXL1、TET2是MN-LGLP最常见的伴随基因突变，伴有STAG2突变MN-LGLP患者预后较差。

## Abstract (en)

Myeloid neoplasms（MNs）belong to a group of hematological malignancies characterized by the abnormal biological functions of hematopoietic stem progenitor cells. The abnormal immune and hematopoietic microenvironment of patients with MN interact with malignant clonal hematopoietic stem cells, promoting the occurrence and development of their diseases. MN large granular lymphocyte proliferation（MN-LGLP）is a special and rare clinical phenomenon in this type of disease. Currently, research on this disease in domestic and international cohorts is limited. This study analyzes the clinical and laboratory characteristics of this type of patient and explores the impact of LGLP on the clinical characteristics and survival of patients with MN. Patients with MN-LGLP are prone to neutropenia and splenomegaly. The presence of LGLP is not a risk factor affecting the survival of patients with MN-LGLP. STAG, ASXL1, and TET2 are the most common accompanying gene mutations in MN-LGLP, and patients with MN-LGLP and STAG2 mutations have poor prognoses.

髓系肿瘤（Myeloid neoplasms，MN）为一组造血干祖细胞生物学功能异常的血液系统恶性肿瘤，主要包括急性髓系白血病（AML）、骨髓增生异常综合征（MDS）以及骨髓增殖性肿瘤（MPN）。越来越多的证据表明，MN患者异常的免疫、造血微环境与恶性克隆性造血干细胞相互作用，促进其疾病的发生、发展[1]–[3]。大颗粒淋巴细胞（LGL）是一组具有不同形态的异质性淋巴细胞群体，其参与先天免疫和免疫监视。LGL起源于两大细胞系：约85％为CD3^+^ T淋巴细胞（T-LGL）[4]；10％～20％为CD3^−^NK细胞（NK-LGL）[4]–[5]。LGL增多可表现为轻度淋巴细胞增多、无症状的多克隆LGL增多、单克隆LGL增多，甚至进展为大颗粒淋巴细胞白血病（LGLL）[6]，其中，无症状的多克隆LGL增多、单克隆LGL增多归为大颗粒淋巴细胞增殖（Large granular lymphocytic proliferation，LGLP）。目前国内对MN-LGLP仅有少量病例报道，尚无队列研究[7]–[10]。本研究分析16例伴有LGLP的MN患者与年龄性别匹配不伴有LGLP的MN患者临床特征、基因突变情况及生存情况，为探索LGLP对MN发生、发展的作用，提供理论基础。

## 病例与方法

1. 病例：纳入279例2017年7月至2022年6月于天津医科大学第二医院确诊的MN患者（AML 61例，MDS 67例，MPN 151例），所有患者均应用流式细胞术免疫分型、TCR基因重排等方法检测LGL及其克隆表达情况。16例诊断为MN-LGLP，其中T-LGLP 14例，NK-LGLP 2例。选取年龄、性别匹配的不伴有LGLP的MN患者，分析两组患者的临床及分子生物学资料。MN诊断符合2016年WHO诊断标准。本研究获得天津医科大学第二医院伦理委员会批准（批件号：KY2023K113），所有研究参与者均按照《赫尔辛基宣言》的要求签署知情同意书。

2. 流式细胞术免疫分型：LGL亚型由流式细胞术分析确定，NK-LGL为CD3^−^/CD56^+^，T-LGL为CD3^+^CD57^+^，样本分析至少研究了以下标志物：CD3/CD8/CD56/CD57。使用TCR Vβ24试剂盒进行T-LGL克隆性分析，将单个KIR抗原（CD158a、CD158b或CD158e）的表达作为NK-LGL克隆性指标。所有抗体及试剂盒来自于美国BD及Beckman Coulter公司，应用Beckman流式细胞仪进行检测，使用Beckman Coulter公司Kluza软件进行数据分析。

3. TCR基因重排：取患者骨髓（EDTA抗凝），提取单个核细胞基因组DNA。采用PCR体外扩增的方法，PCR引物的5′端标记荧光染料，采用毛细管电泳对扩增产物片段进行分离，通过荧光检测系统确定扩增片段的长度和荧光信号高度，判断检测的标本是否存在发生克隆重排的细胞群体。从正常或者多克隆细胞群扩增V-J区域得到毛细管电泳图，通过数据处理，分析TCR基因重排结果。

4. LGLP诊断标准：至少满足以下2条标准：①外周血中流式细胞术免疫表型检测LGL比例>30％；②T细胞受体（TCR）经PCR检测证实为单克隆重排；③骨髓中存在LGL浸润。

5. 染色体核型：采用G或R显带。每例患者至少分析20个中期分裂象，依据《人类细胞遗传学国际命名体制（ISCN2013）》[11]标准进行核型描述。

6. 二代测序：取患者骨髓标本提取DNA，并构建插入片段为200 bp的文库，采用高效液相探针捕获平台捕获目标序列），采用Illumina HiSeq X二代测序平台，对文库进行双末端测序，平测序长度为2×150 bp。测序后以GRCh37.p13为参考基因，结合dbSNP、COSMIC、SIFT、ClinVar等公认数据库进行生物信息学分析。选择了325个基因为目标，确定其致病基因的突变位点。

7. 治疗：16例MN-LGLP患者中，真性红细胞增多症（PV）-LGLP患者应用干扰素治疗，原发性骨髓纤维化（PMF）-LGLP患者采用芦可替尼治疗，4例白血病患者均应用常规化疗。10例MDS-LGLP患者中，3例患者予输血等支持治疗，余7例应用去甲基化药物治疗。

8. 随访：通过查阅门诊/住院病历及电话随访的方式获取患者生存情况。随访截止日期为2023年5月31日，中位随访时间为20（0.5～61）个月，所有患者均未失访。总生存（OS）时间定义为MN患者确诊LGLP至任何原因死亡的时间。

9. 统计学处理：采用SPSS 23.0和R 4.1.2软件进行数据分析。定性资料比较采用配对*t*检验和Fisher's检验，分组比较采用Mann-Whitney *U*检验和Kruskal-Wallis检验。生存分析采用Kaplan-Meier分析方法，*P*<0.05为差异有统计学意义。

## 结果

1. MN-LGLP患者临床和实验室特征：对16例MN-LGLP患者的外周血进行了流式细胞术免疫分型检测。15例外周血LGL占淋巴细胞比例均>30％，1例患者外周血检测CD3^−^CD56^+^ NK-LGL占淋巴细胞比例为21.12％，但存在骨髓LGL浸润及TCR重排阳性。应用PCR检测TCR基因重排，有11例患者显示β和（或）γ基因重排，有5例显示基因重排阴性。骨髓流式细胞术免疫分型检测提示10例患者存在单克隆大颗粒细胞浸润。4例（25％）患者存在染色体异常，7例（43.8％）患者出现脾大（表1）。

**表1:** 16例髓系肿瘤伴大颗粒淋巴细胞增殖患者一般特征

例号	性别	诊断	年龄（岁）	HGB（g/L）	PLT (×10^9^/L)	WBC (×10^9^/L)	ANC (×10^9^/L)	LGL (×10^9^/L)	外周血LGL百分比（%）	LGL骨髓浸润（%）	脾大	染色体核型	TCR重排
1	女	PV-MF	63	113	86	12.1	4.10	0.64	36.65	1.10	有	47, XX, +9	（−）
2	男	PMF	70	48	60	2.02	1.12	0.18	31.19	5.76	有	46,XY	(−)
3	男	AML-M_5_	74	70	111	34.91	0.98	8.05	41.71	阴性	无	46, XY	TCRδ(+)
4	女	AML-M_5_	62	67	94	2.5	0.40	0.29	33.37	4.92	有	46, XX	(−)
5	男	AML-M_5_	79	111	74	1.35	0.13	0.29	34.22	阴性	无	47，XY,+8	TCRβ(+)
6	男	AML-M_3_	77	81	3	0.84	0.12	0.16	21.12	30.11	无	46，XY	TCRβ(+)
7	男	MDS-EB1	89	60	7	0.67	0.10	0.15	30.87	1.47	有	46,XY,del(20q)	(−)
8	男	MDS-EB1	83	58	71	2.1	1.32	0.22	35.67	8.58	无	46, XY	TCRδ(+)
9	男	MDS-RS	68	72	285	2.9	1.79	0.37	36.75	阴性	无	46, XX	TCRβ、δ(+)
10	女	MDS-EB1	61	110	89	1.19	0.25	0.22	33.62	8.68	无	46, XX	TCRβ、δ(+)
11	女	MDS-SLD	51	80	71	1.57	0.85	0.14	30.97	阴性	有	47, XY,+8	TCRδ(+)
12	女	MDS-EB2	59	58	16	1.63	0.18	0.37	31.86	2.92	无	46, XX	(−)
13	女	MDS-EB1	67	93	22	0.27	0.01	0.08	35.14	47.74	无	46, XX	TCRβ(+)
14	女	MDS-EB1	69	92	99	1.8	0.80	0.29	30.41	阴性	无	46, XX	TCRβ(+)
15	女	MDS-MLD	45	126	39	1.66	0.42	0.31	39.44	阴性	有	46, XX	TCRβ(+)
16	男	MDS-EB1	56	143	66	3.7	0.95	0.97	32.94	3.98	有	46, XY	TCRβ(+)

**注** PV-MF：真性红细胞增多症继发骨髓纤维化；PMF：原发性骨髓纤维化；AML：急性髓系白血病；MDS-EB：骨髓增生异常综合征（MDS）伴原始细胞增多；MDS-RS：MDS伴环状铁粒幼红细胞；MDS-SLD：MDS伴单系血细胞发育异常；MDS-MLD：MDS伴多系血细胞发育异常；ANC：中性粒细胞绝对计数；LGL：大颗粒淋巴细胞

将16例MN-LGLP患者与年龄、性别匹配的64例MN患者进行比较发现，MN-LGLP患者中性粒细胞绝对计数（ANC）明显低于对照组（*P*＝0.033）（表2），且MN-LGLP患者发生脾大的比例更高（*P*＝0.023）。对比10例MDS-LGLP患者与40例MDS患者发现，MDS-LGLP患者的WBC低于对照组（*P*＝0.050）。

**表2:** MN-LGLP与MN患者临床和实验室特征对比

特征	MN-LGLP（16例）	MN（64例）	*P*值
性别（例，男/女）	8/8	29/35	1.000
年龄[岁，*M*（范围）]	67.5（40~89）	64（7~90）	0.166
HGB[g/L，*M*（范围）]	80.5（48~143）	73.5（26~147）	0.318
HGB<110 g/L[例（%）]	11（68.6）	50（78.1）	0.161
HGB<60 g/L[例（%）]	4（25.0）	9（14.1）	0.588
WBC[×10^9^/L，*M*（范围）]	2.02（0.27~34.91）	2.39（0.47~232.00）	0.140
PLT[×10^9^/L，*M*（范围）]	71（3~285）	52（2~972）	0.619
PLT<100×10^9^/L[例（%）]	14（87.5）	46（71.8）	0.622
ANC[×10^9^/L，*M*（范围）]	0.61（0.01~4.10）	1.03（0~113.40）	0.033
ANC<1.5×10^9^/L[例（%）]	14（87.5）	39（60.1）	0.175
ANC<5×10^9^/L[例（%）]	8（50.0）	13（20.3）	0.058
两系减少[例（%）]	10（62.5）	45（70.3）	0.432
三系减少[例（%）]	9（56.3）	30（46.9）	0.701
LDH[IU/L，*M*（范围）]	288（158~444）	325（7.94~5 592）	0.521
β_2_微球蛋白[µg/L，*M*（范围）]	2 390（1 202~6 813）	2 085（286~8 628）	0.691
血栓[例（%）]	3（18.8）	8（12.5）	0.903
脾大[例（%）]	7（43.8）	14（21.9）	0.023

**注** MN-LGLP：髓系肿瘤伴大颗粒淋巴细胞增殖；MN；髓系肿瘤；ANC：中性粒细胞绝对计数

2. 二代测序：在MN-LGLP患者中，STAG2、ASXL1、TET2和U2AF1是最常见的突变基因，突变阳性患者占比为25％、25％、25％和19％。伴STAG2突变的患者具有不易发生ASXL1、TET2的趋势特征，但差异无统计学意义（图1）。对比无LGLP的MN患者，MN-LGLP患者发生STAG2突变比例较高，差异具有统计学意义（25.0％对3.5％，*P*＝0.033）（图2A）。

**图1:**
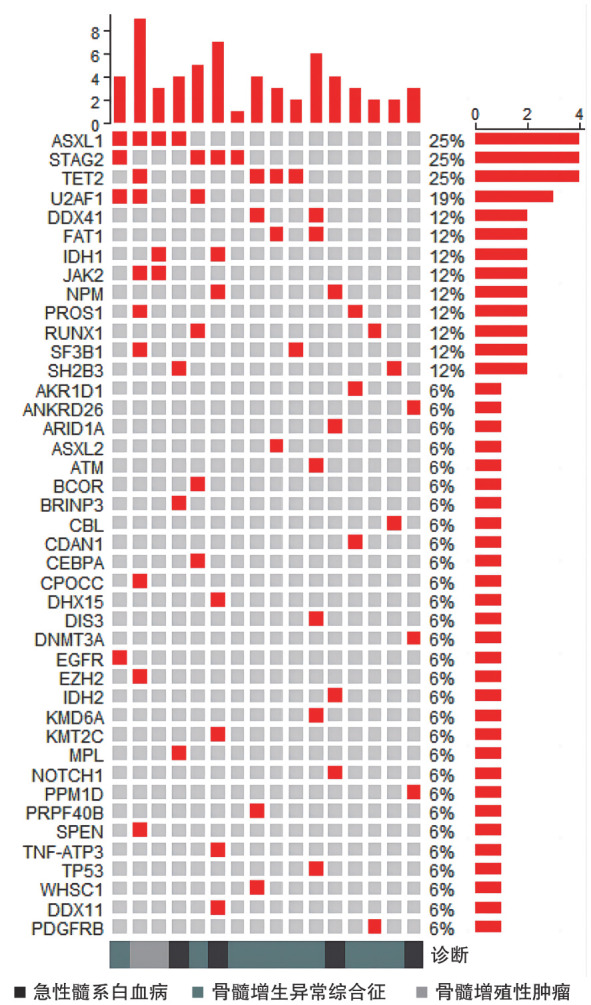
髓系肿瘤伴大颗粒淋巴细胞增殖患者基因突变信息

**图2:**
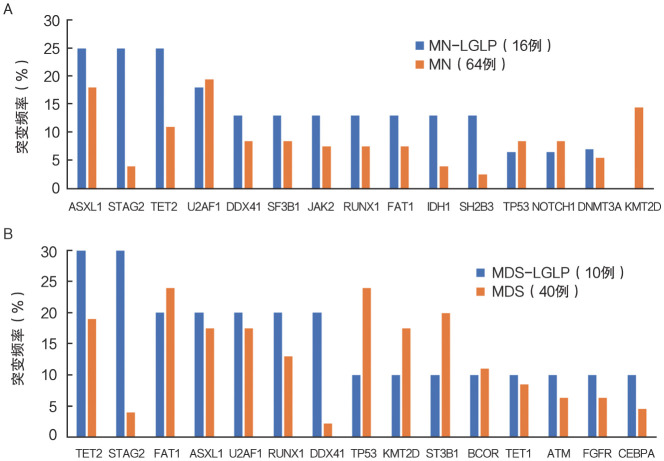
MN-LGLP患者与MN（A）及MDS（B）患者基因主要突变差异 **注** MN-LGLP：髓系肿瘤伴大颗粒淋巴细胞增殖；MN：髓系肿瘤；MDS：骨髓增生异常综合征

进一步分析10例MDS-LGLP患者的二代测序结果发现，STAG2、TET2突变占比最高，均为30％，ASXL1、FAT1、U2AF1、RUNX1、DDX41突变占比均为20％。比较MDS-LGLP与无LGLP的MDS患者，发现MDS-LGLP患者STAG2突变发生比例亦明显高于MDS组（30.0％对2.9％，*P*＝0.034）（图2B）。

3. 生存及预后影响因素分析：截至随访日期，16例MN-LGLP患者中有7例死亡（其中MDS 4例，AML 3例），应用Kaplan-Meier生存分析发现，MN-LGLP和MDS-LGLP患者较年龄、性别匹配的MN、MDS患者均有较好生存的趋势，但差异未达到统计学意义（*P*＝0.270，*P*＝0.450）（图3A、3B）。

**图3:**
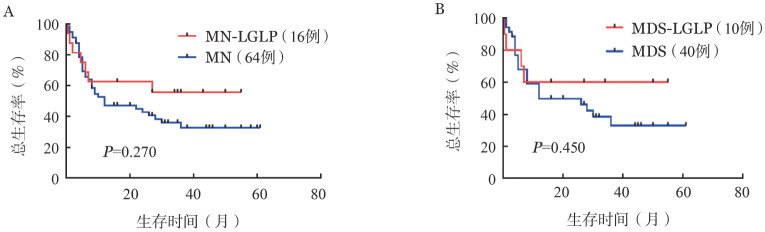
MN-LGLP患者与MN（A）及MDS（B）患者生存比较 **注** MN-LGLP：髓系肿瘤伴大颗粒淋巴细胞增殖；MN：髓系肿瘤；MDS：骨髓增生异常综合征

根据STAG2突变状态将MN-LGLP分别为两组，比较两组生存发现伴STAG2突变组患者生存明显差于无STAG2突变患者（*P*＝0.020）（图4A）。根据STAG2突变状态将MDS-LGLP分别为两组，比较两组生存发现伴STAG2突变组患者生存明显差于无STAG2突变患者（*P*＝0.020）（图4B）。

**图4:**
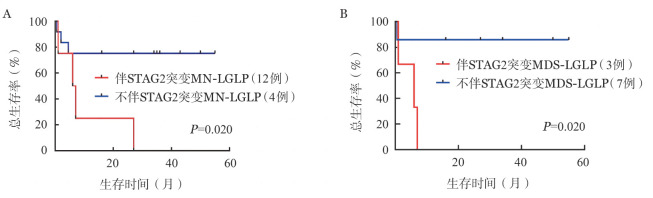
髓系肿瘤（MN）伴大颗粒淋巴细胞增殖（LGLP）（A）和骨髓增生异常综合征（MDS）伴LGLP（B）是否发生STAG2突变患者生存差异

## 讨论

不同于LGLL，LGLP是一类特殊的大颗粒淋巴细胞增殖现象，可继发于各种疾病诱因的反应性增生甚至克隆性扩增[12]。LGLP在MN疾病的发生机制尚不明确，Posnett等[13]研究表明，抗原长期刺激免疫系统，导致细胞毒性T细胞由多克隆增殖逐步转变为寡克隆及单克隆增殖或为本病发病原因。

研究表明，1.38％～50％的MDS患者可合并发生LGLP[9]。本研究MDS-LGLP发生率为21.3％。与既往结果相似，MDS-LGLP患者更多的表现为中性粒细胞减少（90％）、血小板减少、贫血和脾大[10],[14]–[16]。石茵等[10]报道了1例原发性血小板增多症（Primary thrombocytosis，ET）伴T-LGL增殖的病例，该患者TCR基因重排阴性，伴有JAK2^V617F^和DNMT3A突变。在本研究中2例诊断为MPN-LGLP的患者，TCR基因重排亦为阴性，但该2例MPN患者同时伴有JAK2^V617F^和ASXL1突变。JAK2^V617F^、ASXL1/DNMT3A双突变是否与MPN-LGLP发病相关，亦或是偶然现象还有待验证。Malani等[14]报道的AML合并T-LGLL患者初诊时WBC减低，不伴肝脾肿大。在本研究中4例患者为AML-LGLP，初诊时均存在中性粒细胞减少，诱导缓解治疗1周期后复查LGL稳定存在。尽管没有统计学意义，本研究发现MN-LGLP患者具有较好生存的趋势，提示LGLP可能是MN良好生存的保护性因素。

与Ai等[15]和Komrokji等[16]等报道相似，本研究发现，STAG2基因突变在MN-LGLP患者中发生率最高。同时发现，伴STAG2突变的MN-LGL尤其是MDS-LGL患者生存较差，且伴有STAG2突变的MN-LGLP患者不易伴发TET2、ASXL1突变。STAG2为黏连蛋白复合物的核心成分，是一种多聚体蛋白质复合物，为DNA分子周围形成的环状结构，在真核基因组的空间组织中起着多种关键作用。黏连蛋白参与几种基本的细胞功能，包括染色单体凝聚、染色质环组织构成、转录激活以及DNA复制和损伤修复等，其突变导致以上功能缺失，影响了造血干细胞和祖细胞自我更新和分化[17]–[19]。国际上多个多中心的临床研究证实，80％的MDS患者携带至少一种以上的表观遗传学相关的基因突变，其中STAG2、TP53multi BCOR、MLLPTD、BCORL1、FLT3、CEBPA、SF3B15q、ETNK1、NPM1、GATA2、RUNX1、NRAS、IDH1等，作为MDS预后不良的标志[20]–[21]。Tazi等[22]研究也表明，在AML患者中，STAG2突变的长期存在使得染色质结构改变，导致疾病的难治及复发。Karantanos等[23]报道了在MPN患者中STAG2突变与其AML/MDS转化相关。MN-LGLP患者STAG2与其他基因的相关性未见报道。本研究结果提示STAG2突变可能克服LGLP对MN的免疫保护作用，导致MN患者的不良预后。发生在MN-LGLP患者中的基因互斥趋势的机制也需要我们进一步探索。

综上所述，LGLP可能是通过抗肿瘤免疫对MN生存的保护性因素。MN患者合并LGLP，更易出现白细胞和中性粒细胞减少、脾脏肿大。MN-LGLP伴有STAG2突变患者具有较差的生存。
